# The neuroprotective effect of hesperidin in NMDA-induced retinal injury acts by suppressing oxidative stress and excessive calpain activation

**DOI:** 10.1038/s41598-017-06969-4

**Published:** 2017-07-31

**Authors:** Shigeto Maekawa, Kota Sato, Kosuke Fujita, Reiko Daigaku, Hiroshi Tawarayama, Namie Murayama, Satoru Moritoh, Takeshi Yabana, Yukihiro Shiga, Kazuko Omodaka, Kazuichi Maruyama, Koji M. Nishiguchi, Toru Nakazawa

**Affiliations:** 1 Department of Ophthalmology and Visual Science, Tohoku University Graduate School of Medicine, Sendai, 980-8574 Japan; 2 Department of Ophthalmic Imaging and Information Analytics, Tohoku University Graduate School of Medicine, Sendai, 980-8574 Japan; 3 Department of Retinal Disease Control, Tohoku University Graduate School of Medicine, Sendai, 980-8574 Japan; 4 Department of Advanced Ophthalmic Medicine, Tohoku University Graduate School of Medicine, Sendai, 980-8574 Japan

## Abstract

We found that hesperidin, a plant-derived bioflavonoid, may be a candidate agent for neuroprotective treatment in the retina, after screening 41 materials for anti-oxidative properties in a primary retinal cell culture under oxidative stress. We found that the intravitreal injection of hesperidin in mice prevented reductions in markers of the retinal ganglion cells (RGCs) and RGC death after N-methyl-D-aspartate (NMDA)-induced excitotoxicity. Hesperidin treatment also reduced calpain activation, reactive oxygen species generation and TNF-α gene expression. Finally, hesperidin treatment improved electrophysiological function, measured with visual evoked potential, and visual function, measured with optomotry. Thus, we found that hesperidin suppressed a number of cytotoxic factors associated with NMDA-induced cell death signaling, such as oxidative stress, over-activation of calpain, and inflammation, thereby protecting the RGCs in mice. Therefore, hesperidin may have potential as a therapeutic supplement for protecting the retina against the damage associated with excitotoxic injury, such as occurs in glaucoma and diabetic retinopathy.

## Introduction

Oxidative stress is a major cause of various neurodegenerative diseases, including Alzheimer’s disease, Parkinson’s disease, and amyotrophic lateral sclerosis^1^. In the retina, oxidative stress is believed to be an important risk factor for age-related macular degeneration, diabetic retinopathy (DR), and glaucoma. A landmark study, the Age-Related Eye Disease Study (AREDS), showed that anti-oxidant supplements had a beneficial effect in these diseases and could slow their progress^2^. More recently, studies have shown that the level of advanced glycation end products (AGEs) in the skin, which can be used as a biomarker of oxidative stress, is associated with the severity of DR^3, 4^. Thus, treatments targeting oxidative stress are an important strategy for managing retinal diseases.

Among retinal diseases related to oxidative stress, glaucoma is a particularly significant cause of visual impairment and blindness worldwide^5^. Glaucoma is an optic neuropathy characterized by the progressive death of retinal ganglion cell (RGC) axons and the irreversible loss of vision^6^. Although elevated intraocular pressure (IOP) is the most important risk factor for glaucoma, in some patients progress continues despite normal IOP, suggesting that IOP-independent factors also influence glaucoma progression^7, 8^. Furthermore, many previous reports have indicated that oxidative stress-associated compounds increase in the eyes and bodily fluids of human glaucoma patients^9–12^, in particular oxidized DNA^13^. In animal models, oxidative stress is associated with RGC death in the retinas of GLAST- or EAAC1-deficient mice^14^, as well as in mice that were subjected to NMDA-induced retinal injury^15^, optic nerve crush^16^, and optic nerve transection^17^. Oxidative stress-related compounds are also present in the eyes of rats with chronic elevated IOP^18^. Moreover, treatment to decrease oxidative stress prevents RGC death in animal models after optic nerve injury^19, 20^. Thus, if oxidative stress also directly induces RGC death in human glaucoma patients, antioxidant molecules may have neuroprotective effects in glaucoma.

Among antioxidant molecules, flavonoids may be a particularly promising source of new treatments. Flavonoids are a group of plant-derived polyphenolic compounds found in many fruits and vegetables^21–23^, and have been reported to confer a variety of physiological benefits, such as protection from cardiovascular diseases and cancer. Insufficient intake of some flavonoids may be associated with a higher risk of Parkinson’s disease and dementia^24^, and the intake of antioxidant flavonoids is inversely related to the incidence of dementia, including dementia associated with Alzheimer’s disease^25^.

Therefore, the objective of this study was to identify antioxidant compounds in food that could most effectively protect the retinal neurons, especially the RGCs. We used a preliminary *in vitro* experiment, based on a retinal primary culture assay, to screen a variety of food-derived compounds. Based on our findings, we performed a follow-up *in vivo* experiment to assess the neuroprotective effect of the most promising compound, hesperidin, in the RGCs after NMDA injury in mice. We chose this animal model because of the well-known ability of NMDA to promote cellular oxidative stress via the NMDA receptors and cause calcium overload in cells^1, 26^.

## Result

### Identification of compounds with an antioxidant effect: hesperidin ameliorates reduced cell viability in retinal primary cultures under oxidative stress

**Figure 1:**
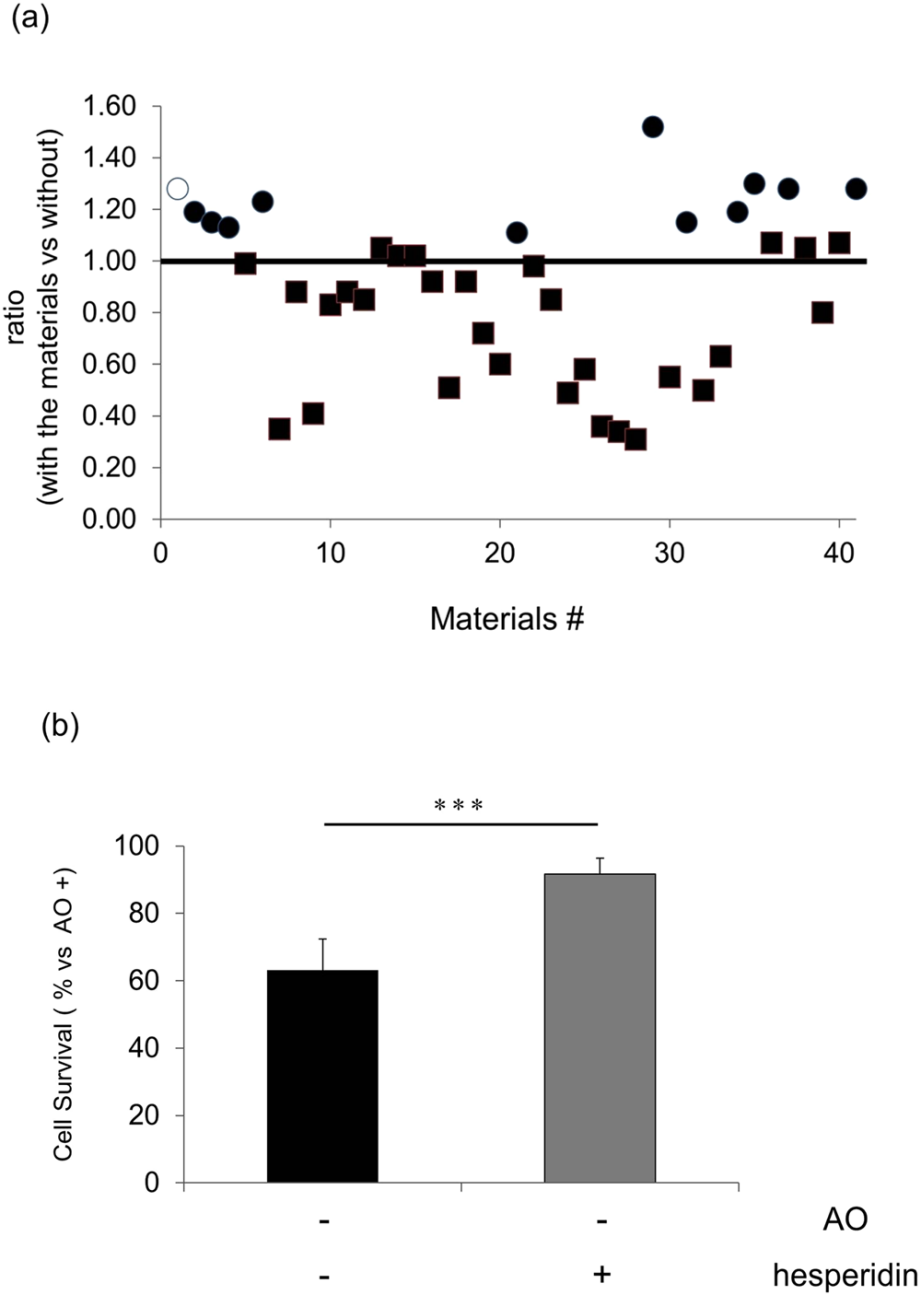
Anti-oxidant compound screening with a mouse retinal primary culture. (**a**) Primary retinal cells were cultured for 18 h under oxidative stress without an antioxidant supplement, in media containing candidate materials. Cell viability was then assessed with an Alamar blue assay. The Y-axis shows the ratio of fluorescence intensity in these cultures, with the intensity of cultures without candidate materials set as 1.0. Individual materials that significantly increased cell viability are represented by black circles (⚫) and materials that did not significantly increase cell viability are represented by squares (◾). Hesperidin is represented by a white circle (⚪). (**b**) Primary retinal cells were cultured with 0.05% hesperidin, and cell viability was assessed with an Alamar blue assay. Fluorescence intensity was measured and the value was normalized as the percentage of the intensity of culture containing an antioxidant supplement. Data represent mean ± SD (*n* = 6 each). ***p < 0.001.

To identify compounds with the potential to protect retinal cells, we evaluated the effect of a variety of test compounds (see Supplementary Table 1) on the viability of primary cultured retinal cells under oxidative stress. To induce oxidative stress in the cultured retinal cells, we excluded 4 anti-oxidant supplements (vitamin E, catalase, vitamin E acetate, superoxide dismutase and glutathione) from a B27 supplement. Viability was assessed with an Alamar blue assay 24 h after the cells were harvested. Twelve candidate compounds significantly improved cell viability in comparison with cell cultures lacking any test compounds (Fig. 1a). However, some of the tested compounds decreased cell viability, suggesting that they might be toxic to the primary retinal cells under the conditions of this study.

Among the 12 candidate compounds with a neuroprotective effect, hesperidin was notable for the strength of its neuroprotective effect (among the top 5; Supplementary Table 1) and good repeatability (Supplementary Figure 1). Taken together with many previous reports showing that hesperidin has anti-apoptotic, anti-oxidative stress and anti-inflammatory effects^27–34^, we thus considered that hesperidin was the best candidate for further study.

An additional experiment showed that the viability of cells in cultures lacking antioxidants was 63.1% of the viability of cells in cultures containing antioxidants (Fig. 1b), and that treatment with 0.05% hesperidin had a significant ameliorative effect, increasing cell viability to 91.7%. Thus, hesperidin ameliorated oxidative stress-induced retinal cell damage *in vitro*.

### Hesperidin ameliorates reductions in RGC markers after NMDA-induced retinal damage

**Figure 2:**
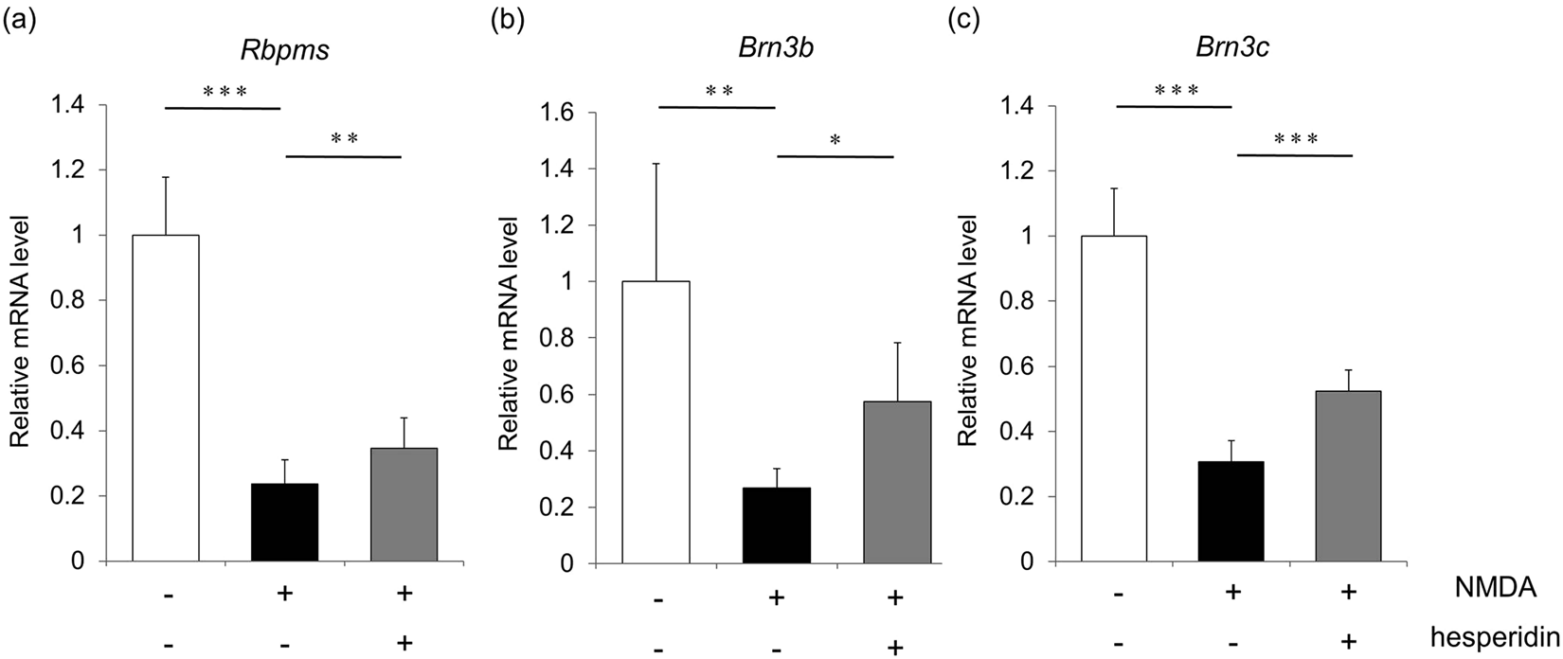
Gene expression of RGC markers in NMDA-injured mouse retinas with and without hesperidin treatment. The transcriptional levels of Rbpms (**a**), Brn3b (**b**) and Brn3c (**c**) were compared in the eyes of mice treated with PBS without NMDA (white bars), treated with PBS (black bars), and treated with hesperidin (gray bars) 24 h after NMDA injection. The gene expression of each RGC marker was normalized to Gapdh. Expression levels are shown as percentages of the average expression in eyes treated with PBS without NMDA. Data represent mean ± SD (Rbpms: n = 14, Brn3b, Brn3c: n = 6 each). *p < 0.05, **p < 0.01, ***p < 0.001.

To evaluate whether hesperidin could protect against RGC damage in the eyes of mice *in vivo*, we measured the gene expression of various RGC markers with real-time RT-PCR. First, to determine the optimal concentration of hesperidin for protecting the RGCs, we injected the mice with 2 μl/eye of 15 μM NMDA intravitreously, without hesperidin or with 0.17%, 1.7% or 17% (w/v) of hesperidin. We then evaluated the effect of these injections on the gene expression of RGC markers. This experiment showed that a concentration of 17% hesperidin was the most effective at suppressing the reduction of RGC markers (Supplementary Figure 2). In addition, we found that NMDA treatment lowered the expression of specific markers of the RGCs, including RNA binding protein with multiple splicing (Rbpms), Brn3b and Brn3c^35, 36^, in comparison with PBS-treated retinas. Hesperidin treatment in the NMDA-treated retinas significantly mitigated this reduction (Fig. 2a). Similarly, NMDA treatment lowered the transcriptional levels of Brn3b and Brn3c, and hesperidin treatment significantly mitigated the reduction (Fig. 2b,c). This result suggests that the intravitreal injection of hesperidin reduced excitotoxic damage to the RGCs in mice.

### Hesperidin prevents RGC death after NMDA injury in mice

**Figure 3:**
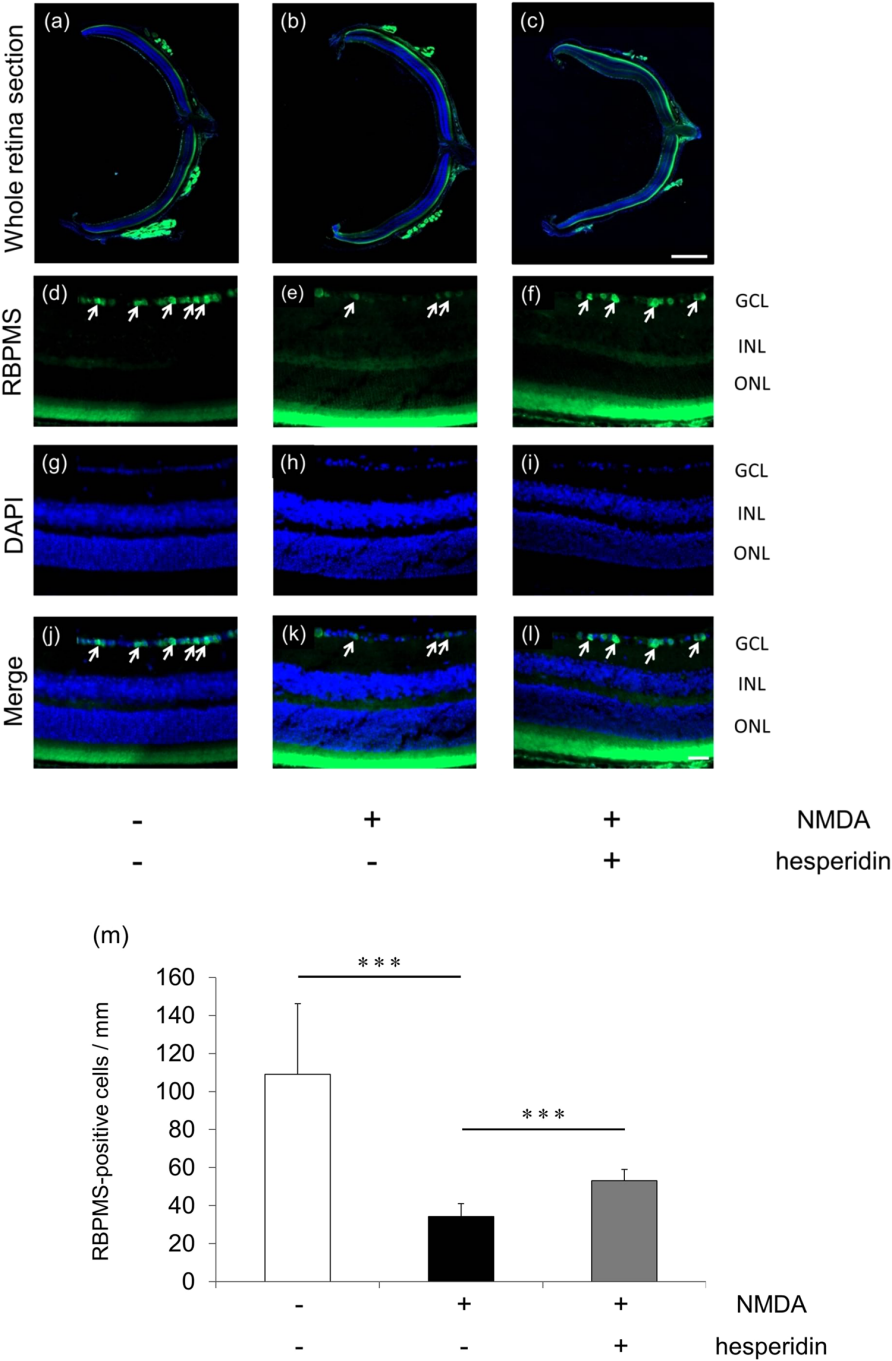
Hesperidin treatment preserved RGC marker-positive cells after NMDA injury. Representative photographs at lower (**a**–**c**) and higher (**d**–**l**) magnification with nuclear staining (DAPI) and immunostaining with anti-RBPMS antibody, taken 24 h after intravitreal injection with PBS (**a**,**d**,**g**,**j**), 30 nmol NMDA (**b**,**e**,**h**,**k**), or NMDA with hesperidin (**c**,**f**,**i**,**l**). RBPMS-positive cells in the GCL are shown by the arrows. (**m**) Histogram showing the average number of RBPMS-positive cells in the retinas. Data represent mean ± SD, (vehicle, *n* = 6, NMDA, *n* = 8, NMDA with hesperidin, *n* = 7) ***p < 0.001. A-C scale bar: 500 µm, D-L scale bar: 50 µm. GCL; ganglion cell layer, INL; inner nuclear layer, ONL; outer nuclear layer.

To evaluate whether hesperidin protected against excitotoxic RGC death in mice, we performed RBPMS immunostaining of the RGCs, in addition to retrograde labeling with Fluorogold (FG). These techniques have been established as effective for determining the survival of RGCs^37, 38^. We found that 24 h after NMDA injury, the number of RBPMS-positive RGCs was lower in the NMDA-treated group than the PBS-treated group (PBS: 109.1 ± 37.2 cells/mm vs. NMDA: 34.4 ± 6.7 cells/ mm). The reduction of RBPMS-positive RGCs after NMDA injury was attenuated after treatment with hesperidin (53.1 ± 5.9 cells/mm) (Fig. 3m).

**Figure 4:**
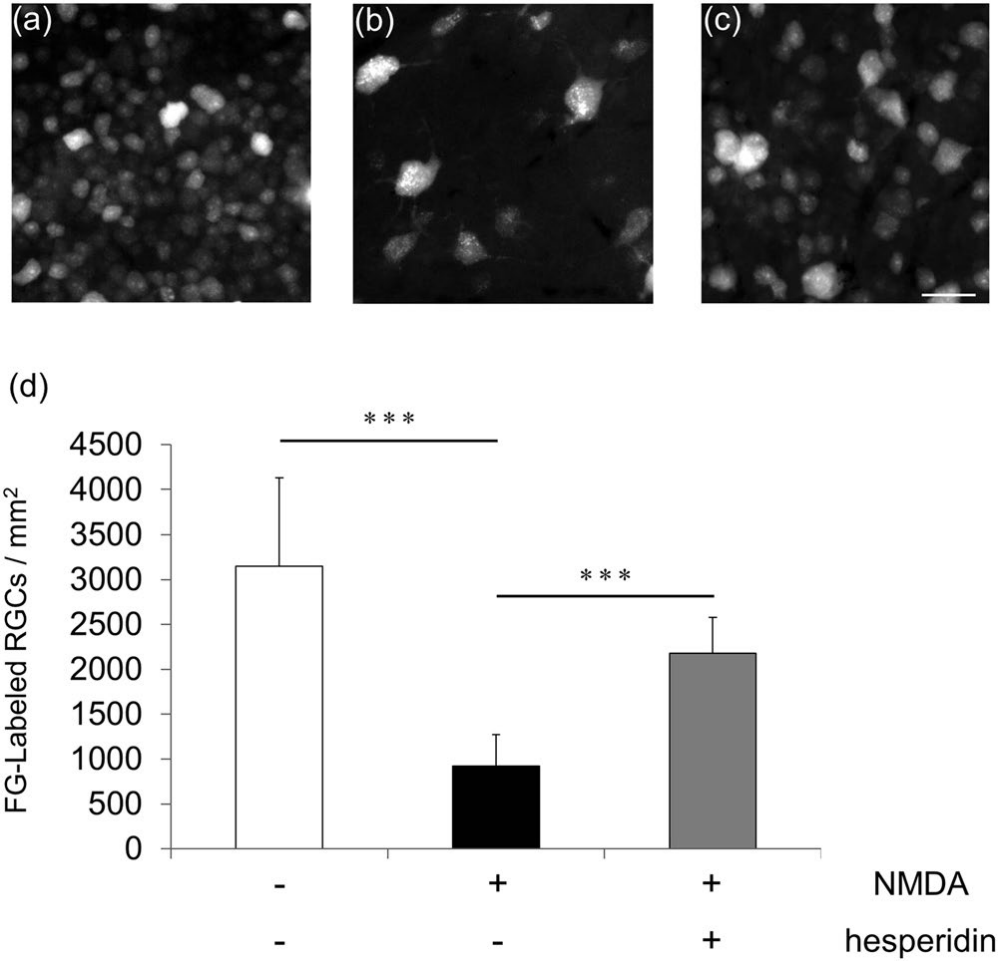
Increase in RGC survival after NMDA injury with hesperidin treatment. Representative images of FG-labeled RGCs 10 days after the intravitreal injection of vehicle (**a**), NMDA (**b**), or NMDA with hesperidin (**c**). Histogram showing the average number of FG-positive cells in each group (**d**). Data represent mean ± SD (vehicle, *n* = 9; NMDA, *n* = 8; NMDA/hesperidin, *n* = 10). ***p < 0.001. Scale bar: 50 µm.

Ten days after NMDA injection, we extracted the eyes from the animals and counted FG-labeled RGCs in the whole retinas. The pattern of changes was similar to the changes in RBPMS-labeled cells. Again, there were significantly fewer FG-labeled RGCs after NMDA injection than after PBS injection (PBS: 3150 ± 979 cells/mm^2^ vs. NMDA: 922 ± 351 cells/mm^2^), and the number of RGCs was significantly higher after treatment with hesperidin (2177 ± 404 cells/mm^2^) (Fig. 4d). This finding suggests that hesperidin prevented RGC death associated with NMDA-induced retinal damage.

### Hesperidin attenuates NMDA-induced lipid peroxidation in the retina

**Figure 5:**
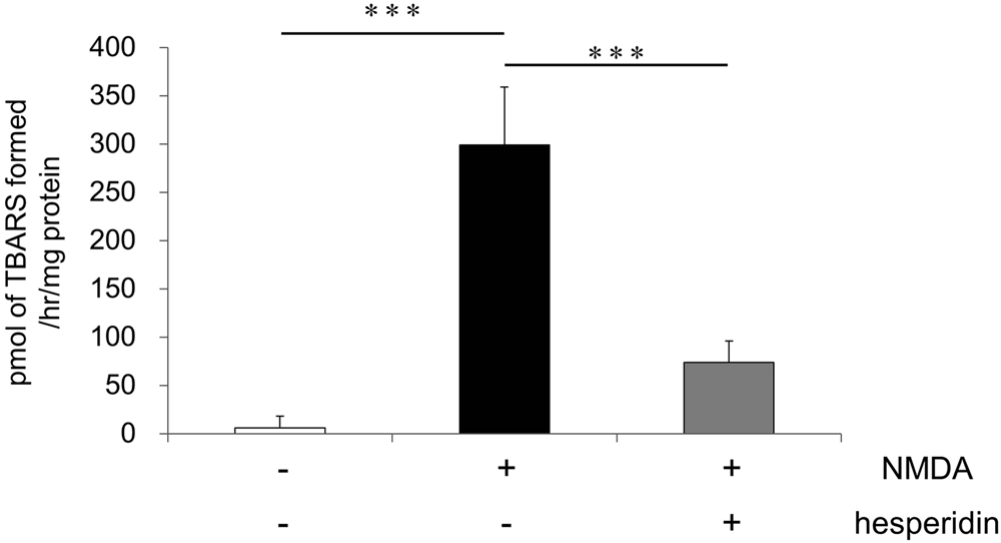
Hesperidin attenuated lipid peroxidation after NMDA injury. The amount of MDA in the retina was quantified using a TBARS assay 6 hours after the intravitreal injection of PBS, NMDA or NMDA with hesperidin. The average amount of MDA was calculated and is shown per mg of retinal protein. (*n* = 6 each) Data represent mean ± SD. ***p < 0.001.

Given previous studies demonstrating the oxidant scavenging function of hesperidin^39^, we next investigated whether hesperidin could ameliorate NMDA-induced oxidative stress. We performed a 2-Thiobarbituric Acid Reactive Substances (TBARS) assay of retinas extracted from mice that had undergone the intravitreal administration of NMDA and hesperidin. This allowed us to quantify the amount of malondialdehyde (MDA). MDA in control retinas injected with PBS was below the detection limit (Fig. 5). By contrast, the amount of MDA in retinas that underwent the intravitreal injection of NMDA was strikingly increased (299.1 ± 59.8 nmol/mg protein; Fig. 5). This finding is consistent with previous reports that NMDA injection leads to increased oxidative stress in the retina^40^. Treatment with NMDA and hesperidin led to a lower level of MDA than treatment with NMDA alone (73.9 ± 22.0 nmol/mg protein, Fig. 5). Thus, we found that hesperidin attenuated NMDA-induced oxidative stress.

### Hesperidin inhibits calpain activation after NMDA treatment in mouse retinas

**Figure 6:**
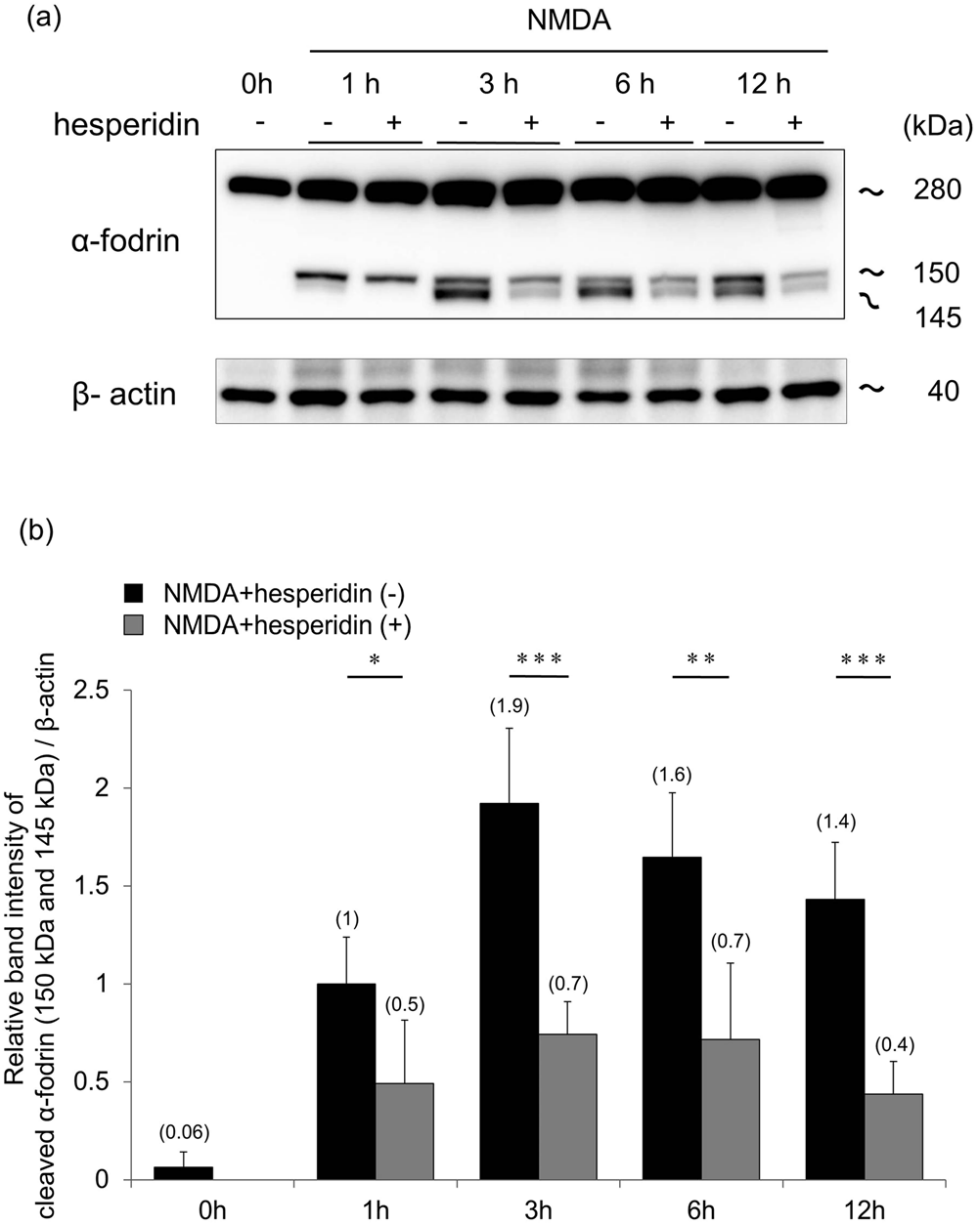
Hesperidin ameliorated cleavage of α-fodrin in the retina after NMDA injury. (**a**) Immunoblot analysis of α-fodrin in retinas with or without hesperidin treatment, 1, 3, 6 and 12 hours after NMDA injury. Representative immunoreaction image with anti-α-fodrin showing intact α-fodrin (280 kDa), caspase-3-cleaved fragmented α-fodrin (150 kDa) and calpain-cleaved fragmented α-fodrin (145 and 150 kDa). β-actin was used as an internal control. (**b**) The relative density of the cleaved-fodrin immunoreactive band. Relative density was based on the cleaved-fodrin immunoreactive band one hour after NMDA injection. Full-length blots are shown in Supplementary Figure 6. Data represent mean ± SD (no treatment and 3 h after NMDA: *n* = 5, other groups: *n* = 6 each). *p < 0.05, **p < 0.01, ***p < 0.001.

Previous studies have shown that the underlying mechanism of RGC death after NMDA treatment involves excess calpain activation, which is induced by elevated oxidative stress^41, 42^. To determine whether hesperidin suppressed excess calpain activation, we analyzed the fragmentation of α-fodrin, an endogenous substrate of calpain^43^, in the retinas of mice after the intravitreous injection of NMDA. Immunoblot analysis showed that the level of cleaved α-fodrin (both the 145 kDa and 150 kDa fragments) was significantly lower with hesperidin treatment than without 1, 3, 6, and 12 hours after NMDA injection (Fig. 6b). Moreover, the fragmentation of α-fodrin was re-analyzed by measurement of the 145 kDa fragments. This showed that it was also significantly lower with hesperidin treatment than without 1, 3, 6, 12 hours after NMDA injection (Supplementary Figure 3). These data show that hesperidin contributed to the suppression of calpain activation after NMDA injury in mouse retinas.

### Hesperidin suppresses the gene expression of pro-inflammatory cytokines in NMDA-treated retinas

**Figure 7:**
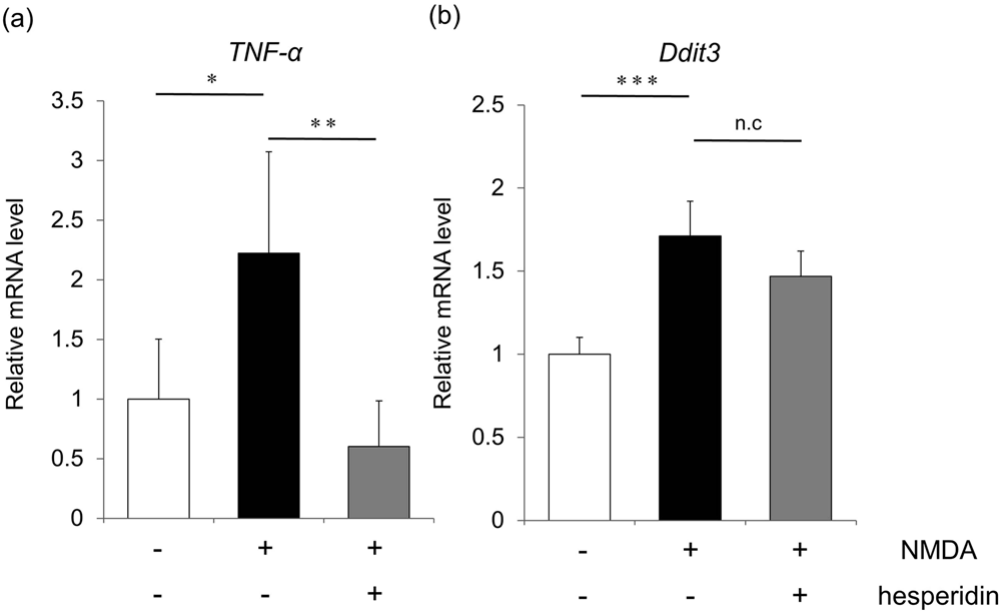
Alterations in the mouse retina in the expression of gene markers for inflammation and ER stress after treatment with NMDA and hesperidin. Damage to the retina after the injection of PBS, NMDA or NMDA/hesperidin was investigated with quantitative RT-PCR measurement of TNF-α (**a**) and Ddit3 (**b**), which are markers of inflammation and ER stress, respectively (n = 6–7). The expression of each gene was normalized to Gapdh. Data represent mean ± SD. *p < 0.05, **p < 0.01.

Past research has shown that TNF-α, a cytokine associated with inflammation, and Ddit3, a protein associated with endoplasmic reticulum (ER) stress, increase in the retina after NMDA injection and cause RGC death^44, 45^. Thus, we used quantitative RT-PCR to investigate whether hesperidin attenuated the expression of the TNF-α and Ddit3 genes after NMDA injury. Without hesperidin treatment, we found that the intravitreal injection of NMDA increased the gene expression of TNF-α to 183% of its expression in animals injected with PBS, and increased the gene expression of Ddit3 to 169%. Animals that received hesperidin treatment in addition to NMDA injection had a significantly lower TNF-α mRNA level, but a statistically similar Ddit3 mRNA level (Fig. 7a,b). This finding suggests that although hesperidin may be able to suppress pro-inflammatory cytokines, such as TNF-α, it cannot suppress ER stress signaling, represented here by Ddit3 expression.

### Electrophysiological evaluation of the RGCs with visual evoked potential

**Figure 8:**
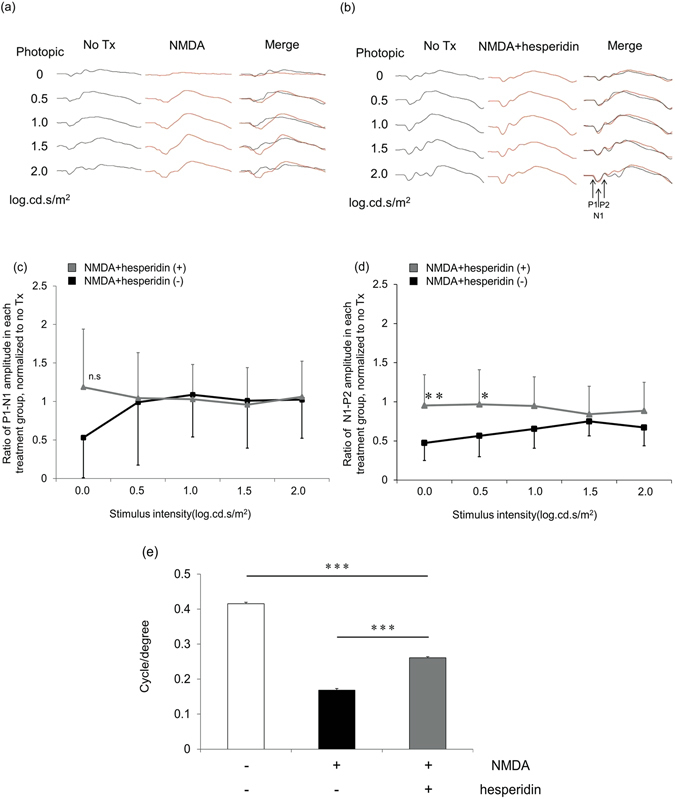
Reductions in visual function after NMDA injury were ameliorated by hesperidin treatment. Representative VEP wave patterns in eyes that were untreated, treated with NMDA, or treated with NMDA and hesperidin (**a**,**b**). P1: initial positive wave, N1: initial negative wave, P2: second positive wave. Graphs showing the amplitude ratio for the P1N1 (**c**) and N1P2 (**d**) waves after treatment with NMDA or NMDA/hesperidin. No treatment: contralateral left eye, NMDA: right eye after NMDA injury (*n* = 10), NMDA/hesperidin: right eye after NMDA and hesperidin injection (*n* = 8). Data represent mean ± SD, *p < 0.05, **p < 0.01. Histogram showing visual acuity (**e**). Visual acuity in the eyes that received NMDA or NMDA/hesperidin injections represents the response to motion in the counterclockwise direction, and visual acuity in the untreated eyes represents the response to motion in the clockwise direction (*n* = 5 each). Data represent mean ± SD, ***p < 0.001.

In order to evaluate the relationship between RGC viability and visual function in mice, we measured the electrophysiological response of the visual cortex, i.e., visual evoked potential (VEP), using a slight modification of a technique that we have previously described^46^. We recorded VEP in waking mice because some anesthetics may act directly or indirectly upon the NMDA receptors^47^. In eyes that were treated with NMDA alone or with NMDA and hesperidin, we evaluated the amplitude ratio of the first positive wave (P1)/first negative wave (N1) and the N1/second positive wave (P2), after normalization to the untreated contralateral eye. We found that the intravitreal injection of NMDA reduced the amplitude ratio of the photopic VEP at a range of stimulus intensities between 0.0 and 2.0 log cd-s/m^−2^. Specifically, the P1-N1 amplitude ratio and the N1-P2 amplitude ratio decreased. This reduction in VEP was partially lessened when the treatment included both hesperidin and NMDA. Hesperidin treatment increased the N1-P2 amplitude ratio by 200% and 170% at stimulus intensities of 0.0 and 0.5, respectively, in comparison with to NMDA injection without hesperidin (Fig. 8d). However, hesperidin treatment had no effect on the decrease in the P1-N1 amplitude ratio (Fig. 8c).

### Optomotry evaluation of ethology

The results of our VEP experiment showed that the intravitreous injection of hesperidin partially ameliorated the cortical changes associated with NMDA-induced RGC injury. Thus, we performed a further experiment to determine if the electrophysiological changes observed at the cortical level had any physiological relevance. We used a technique that we have previously reported, with minor modifications^48^. We assessed visual acuity in NMDA-injured mice with and without hesperidin treatment by measuring the opto-kinetic tracking ability of the animals. We found that the visual acuity of the hesperidin-treated mice (0.42 ± 0.0034 cycle/degree) was better than that of the untreated mice (0.17 ± 0.0089 cycle/degree; Fig. 8e).

## Discussion

In this study, we found that the intravitreal injection of hesperidin prevented excitotoxicity-induced RGC death and improved the electrophysiological and behavioral visual function of mice after NMDA injury. Detailed investigation of the effects of hesperidin treatment showed that it suppressed the effects of oxidative stress both *in vitro* and *in vivo*, while an investigation of the underlying mechanism showed that hesperidin suppressed the over-activation of calpain in NMDA-treated retinas and reduced the up-regulation of TNF-α, an inflammatory cytokine. However, hesperidin had no effect on ER stress.

Dietary supplementation with antioxidants has previously been reported to ameliorate RGC damage caused by glutamate excitotoxicity and oxidative stress in NMDA-treated mice^49^. NMDA is a common choice for animal research in this area because the neuronal death pathways after NMDA injury have been described in detail. In human trials, higher dietary nitrate and green leafy vegetable intake has been shown to be associated with a lower risk of POAG, particularly the risk of POAG with early paracentral visual field loss at the time of diagnosis^50^. These findings suggest that antioxidant compounds present in the diet may have benefits as therapeutic supplements and may help to prevent glaucoma.

Flavonoids are promising candidates for such supplements, because animal studies have shown that they generally have low toxicity at a wide range of doses and lengths of administration. In the current study, we found that among flavonoids, hesperidin was particularly promising. We found that this compound had an antioxidant effect, and that it could improve cell viability in retinal cells *in vitro* and *in vivo*. These findings reinforce previous reports that hesperidin has a vitamin-like activity as an antioxidant^29^, has anti-inflammatory effects^33^, attenuates the activity of caspase-3^27, 28^, attenuates the elevation of intracellular free calcium^30^, improves neuronal energy metabolism^31^, reverses mitochondrial dysfunction^51^, and exerts an antioxidant effect in hepatic L02 cells and PC-12 cells^30–32^. Additionally, hesperidin has been shown to have a preventive effect against neuronal cell death that likely involves the PI3 and MAP kinase pathways^52^. Finally, hesperidin has been shown to reduce excitotoxicity and ROS levels^53^. The current study confirms that hesperidin can reduce oxidative stress and prevent RGC death *in vivo*, suggesting that hesperidin has potential as a new antioxidant supplement to prevent the degeneration of the RGCs in various retinal diseases.

The effect of hesperidin on calpain was a particularly significant finding of this study. Calpain, a member of the cysteine protease family, is activated by an elevated intracellular level of calcium through Ca^2+^ gated ion channels. Calpain modulates cell motility, differentiation, proliferation, apoptosis, signal transduction and gene expression by causing the limited proteolysis of specific substrates^54, 55^. Apoptotic cell death caused by excess calpain activation occurs via both caspase 3-dependent and -independent pathways. These pathways are augmented by reactive oxygen species (ROS) from the mitochondria and induce the expression of apoptosis-inducing factors^56^. Previous studies have reported that calpain is activated during retinal damage, such as from ischemia-reperfusion injury^57^, axotomy^58^, retinal detachment^59^, and excitotoxic injury^60, 61^. Our previous work has also demonstrated that the over activation of calpain occurs in various experimental models of glaucoma, such as NMDA-induced excitotoxicity, optic nerve crush and axonal damage induced by tubulin destruction^41, 62^. The administration of SNJ-1945, a calpain inhibitor, is highly effective at inhibiting RGC death in these disease models, indicating that over activation of calpain has a critical role in promoting RGC death under pathological conditions. In particular, we found that in retinas that underwent oxidative damage caused by the intravitreous injection of 2,2′-azobis (2-amidinopropane) dihydrochloride (AAPH), a ROS generator that induces oxidative stress, calpain was activated and RGC death increased^42^. This finding showed that oxidative stress played a significant role in calpain activation and RGC death.

In the current study, hesperidin suppressed retinal cell damage under oxidative stress *in vitro*, the generation of lipid oxides *in vivo*, and prevented the over activation of calpain in mouse retinas, resulting in a strong protective effect in the RGCs. Hesperidin likely inhibits calpain activation not only by suppressing oxidative stress, but also by reducing calcium influx through the NMDA receptors, as suggested by previous studies showing that hesperidin lowered intracellular calcium (II)^30^ and reduced ROS levels in H_2_O_2_-induced cytotoxicity^63^. Moreover, we found that hesperidin markedly suppressed NMDA-induced elevation in the gene expression of TNF-α, reinforcing previous work^29^. However, we also found that hesperidin could not suppress NMDA-induced elevated Ddit3 gene expression. This suggests that although hesperidin can suppress cell death pathways involving inflammation, it does not suppress those involving ER stress. Moreover, previous studies have found that calcium influx increased Ddit3 gene expression^64, 65^ and ROS production after staurosporine-induced damage in PC12 cells^66^. This may explain why hesperidin did not inhibit calcium influx, despite its suppressive effect on ROS in the signaling pathway of retinal cell death. Furthermore, to confirm that the neuroprotective effects of hesperidin observed in this study were not due to a reaction between hesperidin and NMDA, we performed an additional experiment in which we injected NMDA and hesperidin separately. This showed that even when hesperidin was injected 10 min after NMDA, it still acted to protect against RGC damage *in vivo* (Supplementary Figures 4 and 5). This result provides additional evidence that the effect of hesperidin does not rely on binding with NMDA.

An additional finding of this study was that VEP was partially preserved in NMDA-injured eyes that were also treated with hesperidin. However, it is unclear whether this protective effect had any functional visual impact in the test animals, although behavioral quantification of visual acuity in the injured animals was 50% higher with hesperidin treatment than without. The relatively large effect on visual acuity was beyond our expectations, considering the small improvement observed in the VEP. It may be possible that visual acuity can improve in behavioral testing with a relatively small number of RGCs, while a greater number are necessary to improve VEP measurements.

In conclusion, the results of this study show that hesperidin administration prevented RGC death and improved both electrophysiological function and visual acuity in a mouse model of NMDA-induced excitotoxicity. We obtained evidence that the mechanism of hesperidin’s protective effect involves the suppression of ROS generation, the inhibition of calpain activation, and the suppression of inflammatory cytokines. Therefore, dietary supplementation with food containing hesperidin also promises to have beneficial neuroprotective effects in retinal excitotoxicity-related diseases, such as diabetic retinopathy and glaucoma.

## Methods

### Animals

C57BL/6 J mice (male, 8–12 weeks old) were purchased from SLC Co. (Shizuoka, Japan). All animals were treated in accordance with the principles presented in the guidelines of the Declaration of Helsinki and its guiding principles in the care and use of animals. The Ethics Committee for Animal Experiments at Tohoku University Graduate School of Medicine approved all experimental procedures, in accordance with the Association for Research in Vision and Ophthalmology.

### NMDA-induced retinal injury and hesperidin treatment

NMDA injury was induced in the animals as previously described^67^. A 15-µM solution of NMDA (Sigma-Aldrich, St. Louis, MO, USA) in phosphate-buffered saline (PBS) was injected intravitreally (2 µl/eye). Hesperidin, including hesperetin, (αG hesperidin PAT-T; Glico, Tokyo) in PBS was mixed with NMDA, and the final concentration of hesperidin was 17% and NMDA was 15-µM. The PBS vehicle was injected as a control. Animals with lens injury or vitreous hemorrhage were excluded.

### Mouse retinal primary cultures

The mouse retinas were incubated at 37 °C for 5 min and maintained in a humidified atmosphere of 5% CO_2_ with a Neural Tissue Dissociation Kit (P) (Miltenyi Biotec, Bergisch Gladbach, Germany). Single cell digestion was performed with Gentl MACS according to the manufacturer’s protocol. Incubation time was as follows: m_brain_01: 3 min, m_brain_02: 5 min, and m_brain_03: 5 min; this was followed by additional incubation for 5 min. Single cells were prepared with a cell strainer (40 µm). Manipulation of the primary retinal cultures was performed as previously described, with minor modifications^44, 62^. Cell density was adjusted to 1.5 × 10^5^ cells/50 µl in each well of a 96-well plate (Falcon, NY, USA), and the cultures were incubated for 15 min in a CO_2_ incubator at 37 °C. The culture medium was neurobasal A (Invitrogen, Carlsbad, CA, USA), containing a B27 supplement without anti-oxidants (NBA/B27AO-, Invitrogen), as well as 1 µg/ml insulin, 2 mM L-glutamate and 12 µg/ml gentamicin. Fifteen minutes after the retinal cells were harvested, hesperidin was applied to the primary retinal cells. Two hours later, Alamar blue (Invitrogen) was added. Eighteen hours later, the fluorescence intensity was measured (560-nm excitation and 590-nm emission) with an absorption spectrometer (Vmax; Molecular Devices, Sunnyvale, CA, USA).

### Quantitative real-time PCR

Total RNA extraction and cDNA synthesis was performed as previously described^68, 69^. Predesigned primers and probes, purchased from Life Tec, were used as follows, with the relevant genes in italics: *Pou4f2* (Mm00454754_s1), *Pou4f3* (Mm04213795_s1), and *Rbpms* (Mm00803908_m1)*, TNF-1a* (Mm00441883_g1), *Ddit3* (Mm00492097_m1) and *Gapdh* (Mm01256744_m1). The data was normalized to an endogenous control (GAPDH mRNA).

### Immunohistochemistry and cell counting

Immunohistochemical analysis was performed as previously described^70–72^. The cryosections were blocked with blocking buffer (10% donkey serum in Tw-PBS) at room temperature for 1 h and incubated with a primary antibody against RBPMS (1:200,﻿ Abcam, Cambridge, UK﻿) at 4 °C overnight. After washing with Tw-PBS, the sections were then incubated with Alexa Fluor 488 conjugated goat anti-rabbit IgG antibody (1:500, Invitrogen) in blocking buffer at room temperature for 1 h. The sections were mounted on Vectashield mounting media with DAPI (Vector Laboratories, Burlingame, CA, USA). Photographs of whole retinal sections were taken through a microscope (Axiovert 200; Carl Zeiss, Berkochen, Germany). RPBMS-positive RGCs in the ganglion cell layer (GCL) were counted in the whole retinal sections in a blind fashion.

### Counting FG-labeled RGCs in flat-mounted retinas

Retinal whole mounts were prepared as previously described^71^. Briefly, three days after the NMDA injection, deep anesthesia was induced in the mice with ketamine (100 mg/kg) and xylazine (9 mg/kg) and FG labeling was then performed by injecting 2 µL of 2% aqueous FG containing 1% dimethylsulfoxide (DMSO) into the superior colliculus, using a Hamilton syringe with a 32-gauge needle. Seven days later, the retinas were dissected, fixed in 4% paraformaldehyde (PFA), flat-mounted onto glass slides and mounted with Vectashield mounting media (Vector Laboratories, Burlingame, CA, USA). The number of FG-labeled RGCs was then counted in 12 distinct areas, and the average value in these 12 areas was used for the statistical analysis, as previously described^71^. Photographs were captured with fluorescence microscopy (Axiovert 200, Carl Zeiss). Counting of the FG-labeled RGCs was conducted in a masked fashion by two independent investigators.

### Immunoblotting

Retinal protein extraction, SDS-PAGE and immunoblot analysis were performed as described previously^70^. Briefly, a BCA protein assay was used to measure the quantity of protein. Protein from the retina was separated with 7.5% polyacrylamide gel (Bio Rad, USA) and transferred to a PVDF membrane (Merck-Millipore, Darmstudt, Germany). After blocking with 2% skim milk, the membrane was incubated in a blocking buffer containing rabbit anti-α-fodrin antibody (1:2000; Abcam) at room temperature for 1 h. The membrane was incubated with HRP-conjugated anti-rabbit IgG (dilution 1:5,000; Sigma). The immunoreactive band was developed with ECL prime (GE Healthcare, WI, USA) and examined with ChemiDoc XRS + (Bio-rad). As an internal control, the membrane was incubated with rabbit anti-β-actin antibody (dilution 1:1000; Sigma) at 4 °C overnight. The density of the immunoreactive band was then determined with a digital scanner and Image J software.

### 2-Thiobarbituric acid reactive substances (TBARS) assay

The retinas of the mice were extracted 6 hours after the intravitreal injection of 15 mM NMDA and/or 17% (w/v) hesperidin in PBS (2 µl/eye). The retinas were then homogenized in RIPA buffer with 0.05% (w/v) butylated hydroxytoluene (BHT; Wako Pure Chemical, Osaka, Japan) and a protease inhibitor cocktail (Cayman Chemical Company, Ann Arbor, MI, USA﻿) using a Bioruptor Sonicator (UCD-300; Tosho Denki, Kanagawa, Japan). Next, the retinal homogenates were centrifuged to obtain the lysates required for the TBARS assay. The TBARS assay was performed following a procedure described previously^73^. The fluorescence of the extract was measured at an excitation wavelength of 530 nm and an emission wavelength of 590 nm, with a fluorescence microplate reader (SpectraMax Gemini; Molecular Devices LLC, Sunnyvale, CA)

### Surgical preparation of electrodes

The mice were anesthetized with a single intraperitoneal injection of medetomidine (0.6 mg/kg; Meiji Seika Pharma Co. Ltd., Tokyo, Japan) and ketamine (36 mg/kg; Daiichi Sankyo Co. Ltd., Tokyo, Japan). A surgical procedure was then performed to implant electrodes for the measurement of VEP^46^. Briefly, the VEP electrodes were placed at the right and left primary visual cortices (3.6 mm caudal to the bregma and 2.3 mm lateral). The negative electrode was placed at the area of the right prefrontal cortex (2.0 mm rostral to the bregma). Three stainless steel pan-head screws (M0.6 × 3.0 mm), used to secure the electrodes, were screwed 1.0 mm into the skull so that the tip made light contact with the brain surface. These screws were then fixed using a cyanoacrylate adhesive (Toagosei Co. Ltd., Tokyo, Japan). Subsequently, a polycarbonate bolt (M3 × 10.0 mm) was glued to the exposed skull caudal to the electrodes. The mice were kept warm on a heating pad during the procedure. After the procedure, the mice were administered a reversal agent, atipamezole (0.35 mg/kg; Meiji Seika Pharma Co. Ltd., Tokyo, Japan).

### Recording of VEP in waking mice

Waking VEP recordings were obtained as previously described^46^. A setup consisting of a ganzfeld dome, an acquisition system (PuREC), and an LED stimulator (LS-100; all from Mayo Corp., Inazawa, Japan) were used. After the mice were dark-adapted for at least 6 h, the pupils were dilated with 2.5% phenylephrine and 1.0% tropicamide eye drops. The head of the mouse was firmly fixed to the restraining device while the mouse was allowed to run freely on a rotating cylinder. The mouse and the device were carefully placed in front of the ganzfeld dome after connecting the positive and negative electrodes. First, scotopic VEPs were recorded with a series of white flashes at intensities of −7.0, −6.0, −5.0, −4.0, −3.0, −2.0, −1.0, and 0.0 log cd-s/m^2^. After light adaptation with a white background light (30 cd/m^2^) for 5 min, photopic VEPs were recorded with white flashes at intensities of 0.0, 0.5, 1.0, 1.5, and 2.0 log cd-s/m^2^. The VEP responses were averaged 100 times for each intensity. The VEP signals were band-pass filtered between 0.3 and 50 Hz.

### Optokinetic head-tracking response

The optokinetic head-tracking threshold (OptoMotry; Cerebral Mechanics Inc., Lethbridge, AB, Canada) is a method of measuring the spatial visual sensitivity of mice. The protocol for its use has been described previously^48^. Briefly, this technique yields independent measures of right- and left-eye acuity, based on the differing sensitivity of the two eyes to a rotating pattern. This difference arises because only motion in the temporal-to-nasal direction evokes a tracking response. As a result, the right eye is more sensitive to counterclockwise rotation, while the left eye is more sensitive to clockwise rotations^74^. In the current study, a single researcher measured the optokinetic head-tracking threshold on three days (9, 10, and 11 days after injection with NMDA and hesperidin).

### Statistical analysis

Statistical analysis was performed with JMP Pro 12 software (SAS Institute Inc.) for Windows. Continuous variables were expressed as means. Statistical comparisons were made with a one-way ANOVA followed by the Student t-test or Dunnett’s test, with Bonferroni correction for multiple comparisons. The significance level was set at P < 0.05 (*).

## Electronic supplementary material

Supplementary Information

## References

[1] Coyle JT, Puttfarcken P (1993). Oxidative stress, glutamate, and neurodegenerative disorders. Science (New York, N.Y.).

[2] A randomized, placebo-controlled, clinical trial of high-dose supplementation with vitamins C and E, beta carotene, and zinc for age-related macular degeneration and vision loss: AREDS report no. 8. *Archives of ophthalmology (Chicago, Ill.: 1960)* **119**, 1417–1436 (2001). 10.1001/archopht.119.10.1417 PMC1462955 11594942

[3] Yasuda M (2015). Relationship of skin autofluorescence to severity of retinopathy in type 2 diabetes. Current eye research.

[4] Hashimoto K (2016). The relationship between advanced glycation end products and ocular circulation in type 2 diabetes. Journal of diabetes and its complications.

[5] Quigley HA (1996). Number of people with glaucoma worldwide. The British journal of ophthalmology.

[6] Weinreb RN, Khaw PT (2004). Primary open-angle glaucoma. Lancet (London, England).

[7] Yokoyama Y (2015). Characteristics of patients with primary open angle glaucoma and normal tension glaucoma at a university hospital: a cross-sectional retrospective study. BMC research notes.

[8] Nakazawa T (2016). Ocular Blood Flow and Influencing Factors for Glaucoma. Asia-Pacific journal of ophthalmology (Philadelphia, Pa.).

[9] Tanito M, Kaidzu S, Takai Y, Ohira A (2012). Status of systemic oxidative stresses in patients with primary open-angle glaucoma and pseudoexfoliation syndrome. PloS one.

[10] Himori N (2016). The association between systemic oxidative stress and ocular blood flow in patients with normal-tension glaucoma. Graefe’s archive for clinical and experimental ophthalmology = Albrecht von Graefes Archiv fur klinische und experimentelle Ophthalmologie.

[11] Alvarado J, Murphy C, Polansky J, Juster R (1981). Age-related changes in trabecular meshwork cellularity. Investigative ophthalmology & visual science.

[12] Ferreira SM, Lerner SF, Brunzini R, Evelson PA, Llesuy SF (2004). Oxidative stress markers in aqueous humor of glaucoma patients. American journal of ophthalmology.

[13] Izzotti A, Sacca SC, Cartiglia C, De Flora S (2003). Oxidative deoxyribonucleic acid damage in the eyes of glaucoma patients. The American journal of medicine.

[14] Harada T (2007). The potential role of glutamate transporters in the pathogenesis of normal tension glaucoma. The Journal of clinical investigation.

[15] Inokuchi Y (2009). Edaravone, a free radical scavenger, protects against retinal damage *in vitro* and *in vivo*. The Journal of pharmacology and experimental therapeutics.

[16] Noro T (2015). Spermidine promotes retinal ganglion cell survival and optic nerve regeneration in adult mice following optic nerve injury. Cell death & disease.

[17] Kanamori A, Catrinescu MM, Mahammed A, Gross Z, Levin LA (2010). Neuroprotection against superoxide anion radical by metallocorroles in cellular and murine models of optic neuropathy. Journal of neurochemistry.

[18] Shareef S, Sawada A, Neufeld AH (1999). Isoforms of nitric oxide synthase in the optic nerves of rat eyes with chronic moderately elevated intraocular pressure. Investigative ophthalmology & visual science.

[19] Levkovitch-Verbin H (2000). RGC death in mice after optic nerve crush injury: oxidative stress and neuroprotection. Investigative ophthalmology & visual science.

[20] Himori N (2013). Critical role of Nrf2 in oxidative stress-induced retinal ganglion cell death. Journal of neurochemistry.

[21] Heim KE, Tagliaferro AR, Bobilya DJ (2002). Flavonoid antioxidants: chemistry, metabolism and structure-activity relationships. The Journal of nutritional biochemistry.

[22] Middleton E, Kandaswami C, Theoharides TC (2000). The effects of plant flavonoids on mammalian cells: implications for inflammation, heart disease, and cancer. Pharmacological reviews.

[23] Ross JA, Kasum CM (2002). Dietary flavonoids: bioavailability, metabolic effects, and safety. Annual review of nutrition.

[24] Gao X, Cassidy A, Schwarzschild MA, Rimm EB, Ascherio A (2012). Habitual intake of dietary flavonoids and risk of Parkinson disease. Neurology.

[25] Commenges D (2000). Intake of flavonoids and risk of dementia. European journal of epidemiology.

[26] Choi DW (1992). Excitotoxic cell death. Journal of neurobiology.

[27] Vauzour D, Vafeiadou K, Rice-Evans C, Williams RJ, Spencer JP (2007). Activation of pro-survival Akt and ERK1/2 signalling pathways underlie the anti-apoptotic effects of flavanones in cortical neurons. Journal of neurochemistry.

[28] Choi EJ, Ahn WS (2008). Neuroprotective effects of chronic hesperetin administration in mice. Archives of pharmacal research.

[29] Menze ET, Tadros MG, Abdel-Tawab AM, Khalifa AE (2012). Potential neuroprotective effects of hesperidin on 3-nitropropionic acid-induced neurotoxicity in rats. Neurotoxicology.

[30] Hwang SL, Yen GC (2008). Neuroprotective effects of the citrus flavanones against H2O2-induced cytotoxicity in PC12 cells. Journal of agricultural and food chemistry.

[31] Huang SM, Tsai SY, Lin JA, Wu CH, Yen GC (2012). Cytoprotective effects of hesperetin and hesperidin against amyloid beta-induced impairment of glucose transport through downregulation of neuronal autophagy. Molecular nutrition & food research.

[32] Chen MC, Ye YY, Ji G, Liu JW (2010). Hesperidin upregulates heme oxygenase-1 to attenuate hydrogen peroxide-induced cell damage in hepatic L02 cells. Journal of agricultural and food chemistry.

[33] Meloni F (1995). Effects of 3′-hydroxyfarrerol (IdB 1031), a novel flavonoid agent, on phagocyte products. Inflammation.

[34] Shimouchi A (2016). Neuroprotective effect of water-dispersible hesperetin in retinal ischemia reperfusion injury. Japanese journal of ophthalmology.

[35] Xiang M (1993). Brn-3b: a POU domain gene expressed in a subset of retinal ganglion cells. Neuron.

[36] Xiang M (1995). The Brn-3 family of POU-domain factors: primary structure, binding specificity, and expression in subsets of retinal ganglion cells and somatosensory neurons. The Journal of neuroscience: the official journal of the Society for Neuroscience.

[37] Berkelaar M, Clarke DB, Wang YC, Bray GM, Aguayo AJ (1994). Axotomy results in delayed death and apoptosis of retinal ganglion cells in adult rats. The Journal of neuroscience: the official journal of the Society for Neuroscience.

[38] Kwong JM, Caprioli J, Piri N (2010). RNA binding protein with multiple splicing: a new marker for retinal ganglion cells. Investigative ophthalmology & visual science.

[39] Javed H (2015). Effect of hesperidin on neurobehavioral, neuroinflammation, oxidative stress and lipid alteration in intracerebroventricular streptozotocin induced cognitive impairment in mice. Journal of the neurological sciences.

[40] Ganapathy PS (2011). The role of N-methyl-D-aspartate receptor activation in homocysteine-induced death of retinal ganglion cells. Investigative ophthalmology & visual science.

[41] Nakazawa T (2009). Calpain-mediated degradation of G-substrate plays a critical role in retinal excitotoxicity for amacrine cells. Journal of neuroscience research.

[42] Yokoyama Y (2014). The role of calpain in an *in vivo* model of oxidative stress-induced retinal ganglion cell damage. Biochemical and biophysical research communications.

[43] Nath R (1996). Non-erythroid alpha-spectrin breakdown by calpain and interleukin 1 beta-converting-enzyme-like protease(s) in apoptotic cells: contributory roles of both protease families in neuronal apoptosis. The Biochemical journal.

[44] Nakazawa T (2007). Pitavastatin prevents NMDA-induced retinal ganglion cell death by suppressing leukocyte recruitment. Journal of neurochemistry.

[45] Awai M (2006). NMDA-induced retinal injury is mediated by an endoplasmic reticulum stress-related protein, CHOP/GADD153. Journal of neurochemistry.

[46] Tomiyama Y (2016). Measurement of Electroretinograms and Visually Evoked Potentials in Awake Moving Mice. PloS one.

[47] Anis NA, Berry SC, Burton NR, Lodge D (1983). The dissociative anaesthetics, ketamine and phencyclidine, selectively reduce excitation of central mammalian neurones by N-methyl-aspartate. British journal of pharmacology.

[48] Nishiguchi KM (2015). Gene therapy restores vision in rd1 mice after removal of a confounding mutation in Gpr179. Nature communications.

[49] Nakajima Y (2008). Coenzyme Q10 protects retinal cells against oxidative stress *in vitro* and *in vivo*. Brain research.

[50] Kang JH (2016). Association of Dietary Nitrate Intake With Primary Open-Angle Glaucoma: A Prospective Analysis From the Nurses’ Health Study and Health Professionals Follow-up Study. JAMA ophthalmology.

[51] Wang DM (2013). Protective effects of hesperidin against amyloid-beta (Abeta) induced neurotoxicity through the voltage dependent anion channel 1 (VDAC1)-mediated mitochondrial apoptotic pathway in PC12 cells. Neurochemical research.

[52] Nones J (2011). TC, E. S. & Gomes, F. C. Hesperidin, a flavone glycoside, as mediator of neuronal survival. Neurochemical research.

[53] Ho SL (2015). Inhibition of beta-amyloid Aggregation By Albiflorin, Aloeemodin And Neohesperidin And Their Neuroprotective Effect On Primary Hippocampal Cells Against beta-amyloid Induced Toxicity. Current Alzheimer research.

[54] Adamec E, Beermann ML, Nixon RA (1998). Calpain I activation in rat hippocampal neurons in culture is NMDA receptor selective and not essential for excitotoxic cell death. Brain research. Molecular brain research.

[55] Wu HY (2004). Critical role of calpain-mediated cleavage of calcineurin in excitotoxic neurodegeneration. The Journal of biological chemistry.

[56] Susin SA (1999). Molecular characterization of mitochondrial apoptosis-inducing factor. Nature.

[57] Sakamoto YR (2000). Involvement of calpain isoforms in ischemia-reperfusion injury in rat retina. Current eye research.

[58] McKernan DP, Guerin MB, O’Brien CJ, Cotter TG (2007). A key role for calpains in retinal ganglion cell death. Investigative ophthalmology & visual science.

[59] Sanvicens N, Cotter TG (2006). Ceramide is the key mediator of oxidative stress-induced apoptosis in retinal photoreceptor cells. Journal of neurochemistry.

[60] Chiu K, Lam TT (2005). Ying Li, W. W., Caprioli, J. & Kwong Kwong, J. M. Calpain and N-methyl-d-aspartate (NMDA)-induced excitotoxicity in rat retinas. Brain research.

[61] Volbracht C (2005). The critical role of calpain versus caspase activation in excitotoxic injury induced by nitric oxide. Journal of neurochemistry.

[62] Ryu M (2012). Critical role of calpain in axonal damage-induced retinal ganglion cell death. Journal of neuroscience research.

[63] Tamilselvam K (2013). Neuroprotective effects of hesperidin, a plant flavanone, on rotenone-induced oxidative stress and apoptosis in a cellular model for Parkinson’s disease. Oxidative medicine and cellular longevity.

[64] Bartlett JD, Luethy JD, Carlson SG, Sollott SJ, Holbrook NJ (1992). Calcium ionophore A23187 induces expression of the growth arrest and DNA damage inducible CCAAT/enhancer-binding protein (C/EBP)-related gene, gadd153. Ca^2+^ increases transcriptional activity and mRNA stability. The Journal of biological chemistry.

[65] Tajiri S (2004). Ischemia-induced neuronal cell death is mediated by the endoplasmic reticulum stress pathway involving CHOP. Cell death and differentiation.

[66] Kruman I, Guo Q, Mattson MP (1998). Calcium and reactive oxygen species mediate staurosporine-induced mitochondrial dysfunction and apoptosis in PC12 cells. Journal of neuroscience research.

[67] Nakazawa T (2005). N-Methyl-D-Aspartic acid suppresses Akt activity through protein phosphatase in retinal ganglion cells. Molecular vision.

[68] Sato K (2016). Topical ocular dexamethasone decreases intraocular pressure and body weight in rats. Journal of negative results in biomedicine.

[69] Fujita K (2015). *In vivo* cellular imaging of various stress/response pathways using AAV following axonal injury in mice. Scientific reports.

[70] Sato K (2013). Receptor interacting protein kinase-mediated necrosis contributes to cone and rod photoreceptor degeneration in the retina lacking interphotoreceptor retinoid-binding protein. The Journal of neuroscience: the official journal of the Society for Neuroscience.

[71] Nakazawa T, Tamai M, Mori N (2002). Brain-derived neurotrophic factor prevents axotomized retinal ganglion cell death through MAPK and PI3K signaling pathways. Investigative ophthalmology & visual science.

[72] Nakazawa T (2002). Comparative expression profiles of Trk receptors and Shc-related phosphotyrosine adapters during retinal development: potential roles of N-Shc/ShcC in brain-derived neurotrophic factor signal transduction and modulation. Journal of neuroscience research.

[73] Lederman M (2014). Degeneration modulates retinal response to transient exogenous oxidative injury. PloS one.

[74] Prusky GT, Alam NM, Beekman S, Douglas RM (2004). Rapid quantification of adult and developing mouse spatial vision using a virtual optomotor system. Investigative ophthalmology & visual science.

