# Supplementary material for: The neuroprotective effect of hesperidin in NMDA-induced retinal injury acts by suppressing oxidative stress and excessive calpain activation
Source: Sci Rep. 2017 Jul 31;7:6885. doi: 10.1038/s41598-017-06969-4 (PMC5537259; doi:10.1038/s41598-017-06969-4)

Supplementary Information

The neuroprotective effect of hesperidin in NMDA-induced retinal injury acts by suppressing oxidative stress and excessive calpain activation

Shigeto Maekawa[1], Kota Sato[1,2], Kosuke Fujita[3], Reiko Daigaku[1], Hiroshi Tawarayama[3], Namie Murayama[1], Satoru Moritoh[1], Takeshi Yabana[1], Yukihiro Shiga[1], Kazuko Omodaka[1,2], Kazuichi Maruyama[1], Koji M Nishiguchi[4], & Toru Nakazawa[1,2,3,4,*]

[1] Department of Ophthalmology and Visual Science, Tohoku University Graduate School of Medicine, Sendai, 980-8574, Japan.

[2] Department of Ophthalmic Imaging and Information Analytics, Tohoku University Graduate School of Medicine, Sendai, 980-8574, Japan.

[3] Department of Retinal Disease Control, Tohoku University Graduate School of Medicine, Sendai, 980-8574, Japan.

[4] Department of Advanced Ophthalmic Medicine, Tohoku University Graduate School of Medicine, Sendai, 980-8574, Japan.

Tohoku University Graduate School of Medicine, Department of Ophthalmology, 1-1 Seiryo, Aoba, Sendai, Miyagi, 980-8574, Japan

Corresponding and requests for materials should be addressed to T.N (email: ntoru@oph.med.tohoku.ac.jp).

TEL +81-22-717-7294, FAX +81-22-717-7298

SUPPLEMENTARY MATERIAL

Supplementary table 1

The materials for anti-oxidant compound screening

Forty one supplements were investigated with an Alamar blue assay. Supplementary Table 1 shows compounds name and the ratio of fluorescence intensity in these cultures, with the intensity of cultures without candidate materials set as 1.0.

Supplementary Figure 1

Primary retinal cells were cultured with 0.01%, 0.025%, or 0.05% hesperidin without an antioxidant supplement (AO-) for 18 h, and cell viability was then assessed with an Alamar blue assay. The fluorescence intensity was measured and normalized as the percentage of the intensity of a culture containing an antioxidant supplement. Data represent mean ± SD (*n* = 4 each). *p <0.05, **p <0.01, n.s: not significant.

Supplementary Figure 2

Gene expression of RGC markers in NMDA-injured mouse retinas after treatment with 0.17%, 1.7%, and 17% hesperidin. The transcriptional levels of Rbpms (a), Brn3b (b) and Brn3c (c) were compared in the eyes of untreated mice (white bar) and mice treated with PBS (black bars) or 0.17%, 1.7%, and 17% hesperidin (gray bars) 24 h after NMDA injection. The gene expression of each RGC marker was normalized to Gapdh. Expression levels are shown as percentages of the average expression in the untreated eyes. Data represent mean ± SD (Rbpms n = 6-8, Brn3b n = 7-8, Brn3c n = 7-8). Statistical comparisons were made with a one-way ANOVA followed by Dunnett's test. *p

<0.05, ***p <0.001. n.s: not significant

Supplementary Figure 3

Cleaved 145-kDa fragments of α-fodrin in the retina after NMDA injury were reduced with hesperidin treatment. The graph shows the relative band density of cleaved 145-kDa fragments of α-fodrin after NMDA injection with and without hesperidin. Relative density was based on the immunoreactive band of cleaved 145-kDa α-fodrin fragments one hour after NMDA injection. Data represent the mean ± SD (no treatment and 3 h after NMDA: $n = 5$, other groups: $n = 6$ each). *p <0.05, **p <0.01.

Supplementary Figure 4

Hesperidin treatment after NMDA injection improved RGC survival. Hesperidin treatment was administered 10 min after NMDA injection, and the gene expression of various RGC markers was assessed with qRT-PCR. The graph shows the transcriptional levels at 24 h of Rbpms (a), Brn3b (b) and Brn3c (c) in untreated eyes (white bar) and eyes treated with PBS (black bars) or hesperidin (gray bars) 10 min after NMDA injection. The gene expression of each RGC marker was normalized to Gapdh. Data represent the mean ± SD (each: n = 8). *p <0.05, **p <0.01, ***p <0.001.

Supplementary Figure 5

The cleavage of α-fodrin in the retina was ameliorated even when hesperidin treatment followed NMDA injection. (a) Immunoblot analysis of α-fodrin in retinas treated with PBS or hesperidin, without NMDA injury and 3 h after NMDA injury. In this experiment, NMDA was injected first, and PBS or hesperidin was administered 10 min later. Representative immunoreaction images for

anti-α-fodrin, showing intact α-fodrin (280 kDa), caspase-3-cleaved fragmented α-fodrin (150 kDa) and calpain-cleaved fragmented α-fodrin (145 and 150 kDa). β-actin was used as an internal control. (b) The relative density of the cleaved-fodrin immunoreactive band 3 h after NMDA injection. Data represent the mean ± SD (each group: *n* = 4; no treatment, treatment of NMDA with PBS or hesperidin). *$p < 0.05$.

Supplementary Figure 6

Original image of immunoblotting in the main figure 6.

Uncropped representative immunoreactivity against a-fodrin (a) and b-actin (b) are displayed and arrows indicate the target molecules. We have defined the band of intact a-fodrin, cleaved a-fodrin and b-actin based on molecular weight.

Supplementary table 1

compounds name	Ratio (with the materials vs without)
Hesperidin 0.05%	1.28
Crocetin 0.0005%	1.19
Boysen berry powder 0.01%	1.15
Tamarindus indica extract powder 0.000125%	1.13
Cranberry 20S(Proanthocyanidin 20%) 0.05%	0.99
Cranberry S(Proanthocyanidin 2-3%) 0.05%	1.23
Camucamu 0.05%	0.35
Ampelopsis extract powder 0.05%	0.88
Rosa roxburghii extract powder 0.05%	0.41
dried pomegranate seed extract powder 0.01%	0.83
Apple polyphenol extract powder 0.01%	0.88
Pyrus malus (apple) fruit powder 0.01%	0.85
Lonicera caerulea powder 0.01%	1.05
Glycyrrhiza powder 0.001%	1.02
cinnamon powder 0.001%	1.02
ginger-H powder 0.001%	0.92
grape seed extract powder 0.025%	0.51
chinese wolfberry extract powder	0.92
purple coneflower powder	0.72
Aronia extract powder(from BGGI)	0.6
Aronia extract powder(from with you)	1.11
fermented grape extract powder	0.98
Punica granatum extract powder	0.85
Lemon balm extract powder	0.49
Engelhardia roxburghiana extract powder	0.58
ginkgo leaf extract F extract powder	0.36

ginkgo leaf extract FJ extract powder	0.34
Green tea extract powder	0.31
Hesperidin H	1.52
cassis polyphenol powder	0.55
CoQ10 extract powder	1.15
Amla extract powder	0.5
Astragalus complanatus R.Br extract powder	0.63
Korean ginseng tea extract powder	1.19
Annatto extract powder	1.3
Turmeric extract powder	1.07
Lactic acid bacterium extract powder	1.28
Lactic acid (Sf) powder	1.05
Lactic acid（La) powder	0.8
Lactic acid(Ls) powder	1.07
Lactic acid(Bl) powder	1.28

Supplementary Figure 1

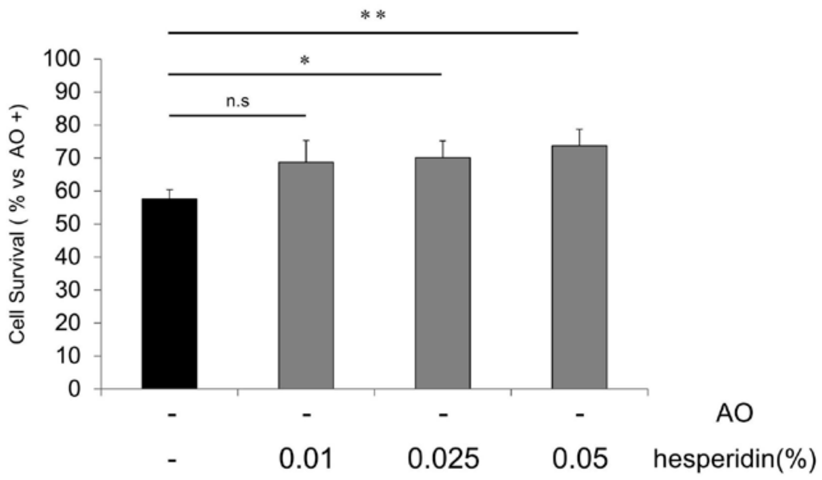

Supplementary Figure 2

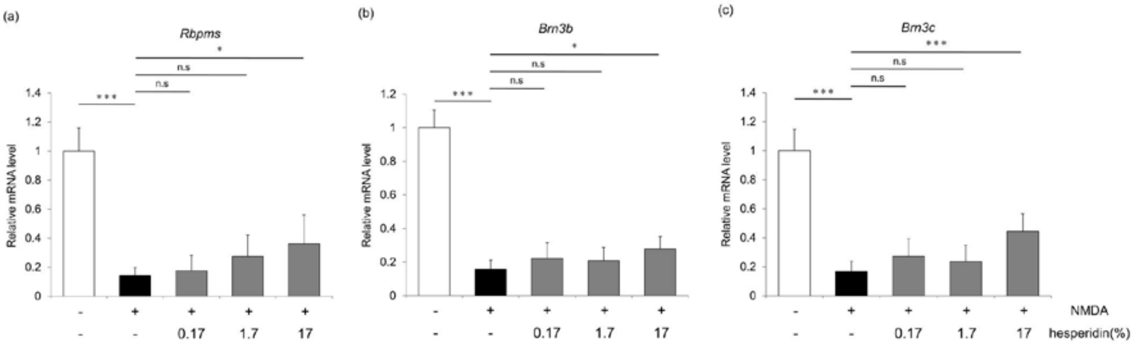

Supplementary Figure 3

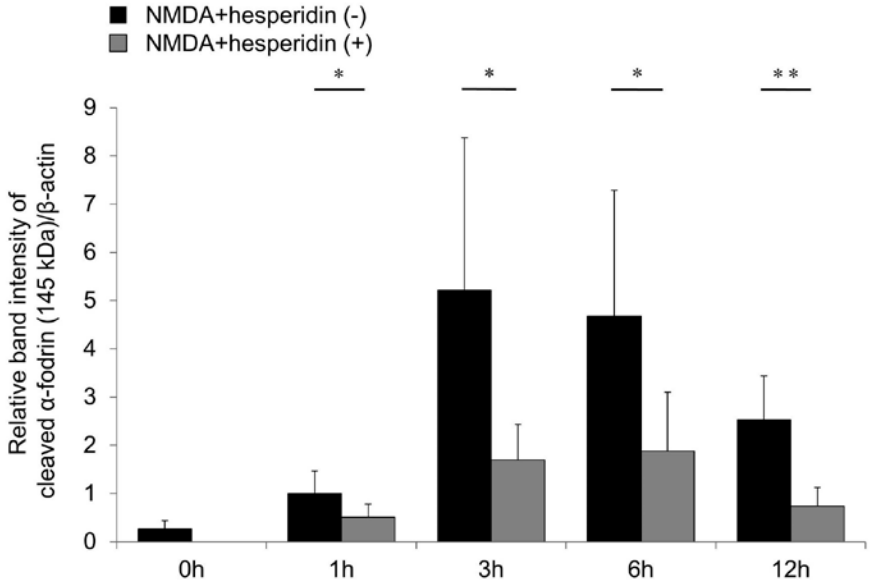

Supplementary Figure 4

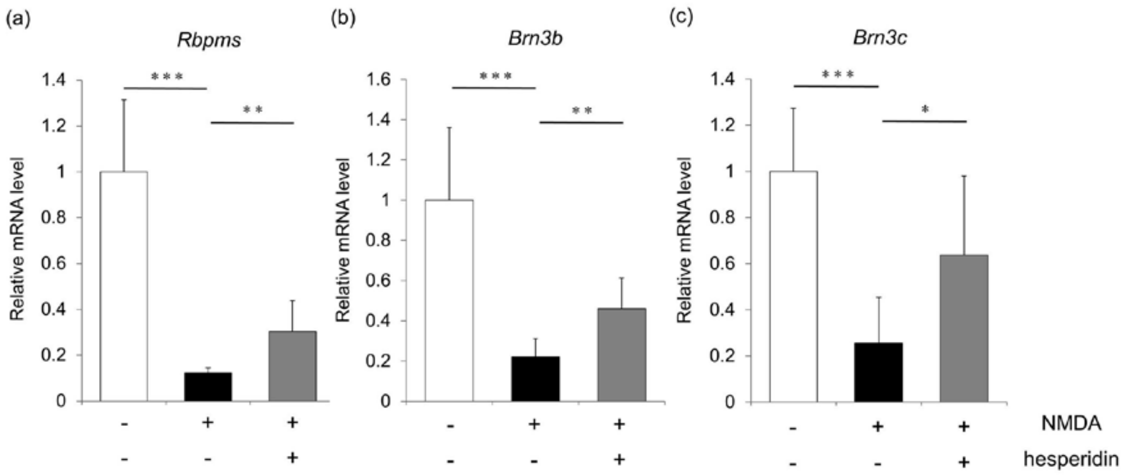

Supplementary Figure 5

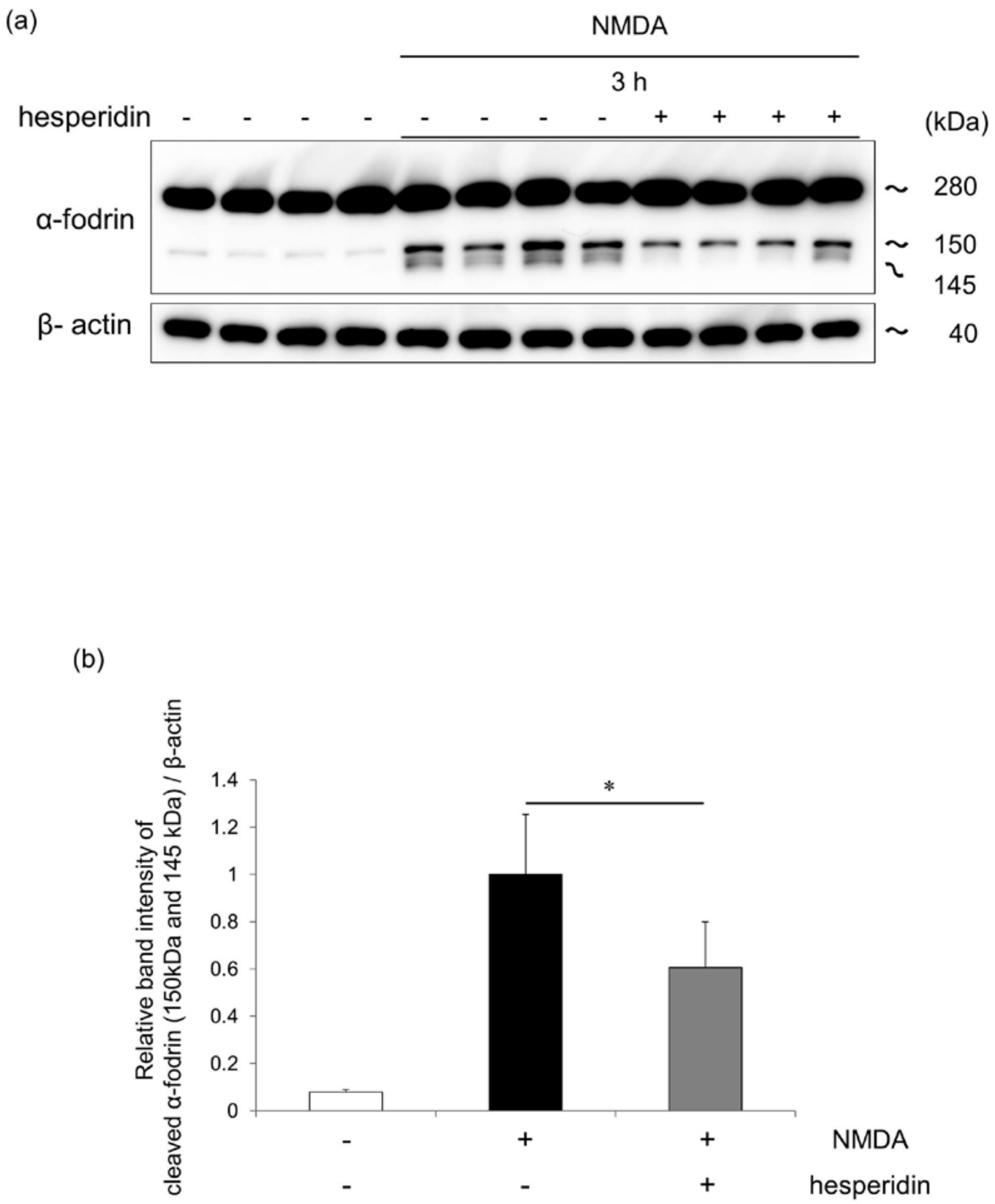

Supplementary Figure 6

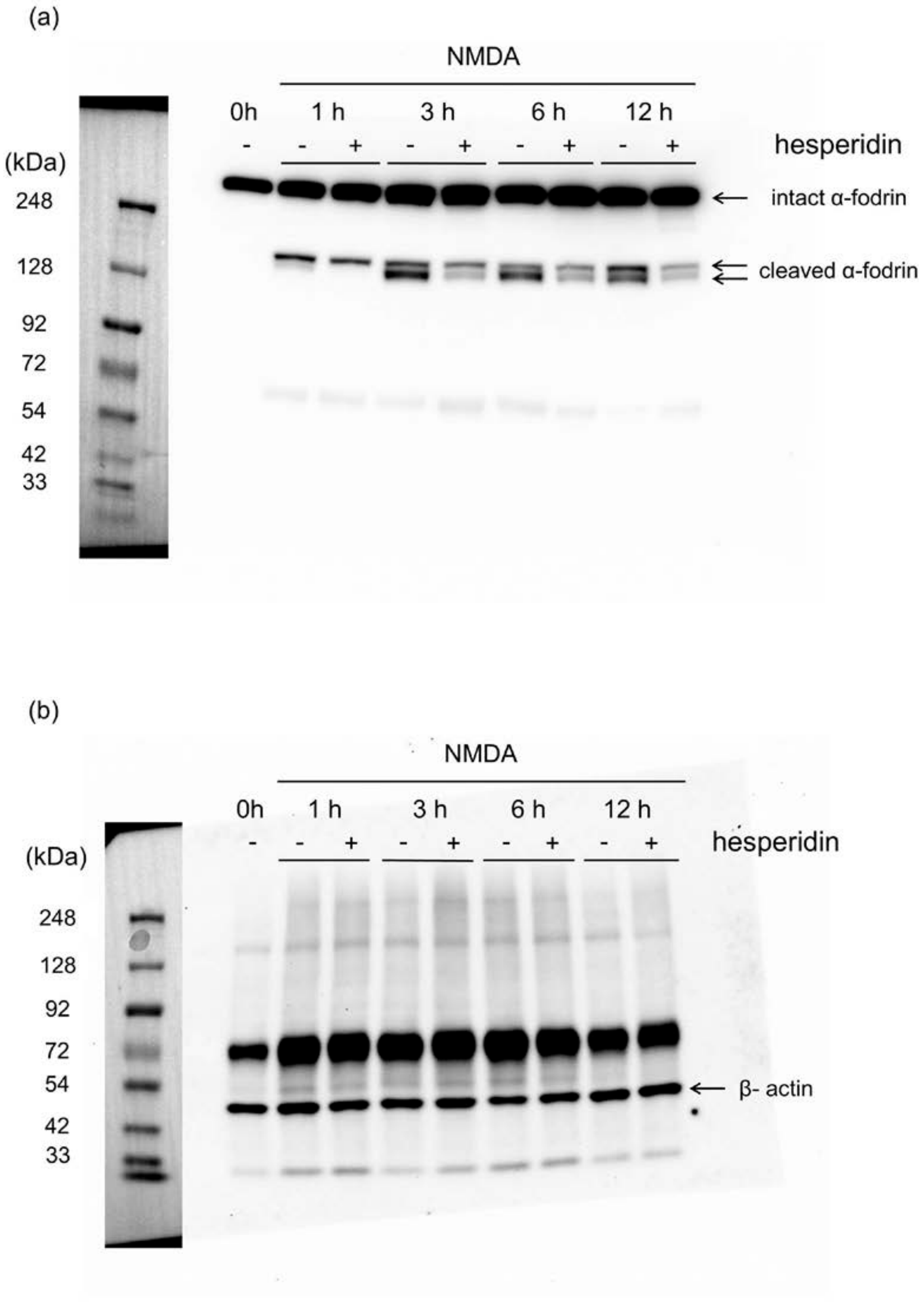

Supplement: Supplementary file 1 — Supplementary Information [file 41598_2017_6969_MOESM1_ESM.pdf]
